# Interfacial Shear Strength of Single-Walled Carbon Nanotubes-Cement Composites from Molecular Dynamics and Finite Element Studies

**DOI:** 10.3390/ma16051992

**Published:** 2023-02-28

**Authors:** Carlos Talayero, Isabel Lado-Touriño, Omar Aït-Salem, Ismael Sánchez Ramos, Alicia Páez-Pavón, Rosario G. Merodio-Perea

**Affiliations:** 1 Engineering Department, School of Architecture, Engineering and Design, Universidad Europea de Madrid, 28670 Villaviciosa de Odón, Spain; 2 Hexagon HMI, 28050 Madrid, Spain

**Keywords:** carbon nanotubes, interfacial shear strength, pull-out test, finite element method, molecular model

## Abstract

Carbon nanotubes (CNTs) are nanometer-sized structures that can be used to reinforce cement matrices. The extent to which the mechanical properties are improved depends on the interfacial characteristics of the resulting materials, that is, on the interactions established between the CNTs and the cement. The experimental characterization of these interfaces is still impeded by technical limitations. The use of simulation methods has a great potential to give information about systems lacking experimental information. In this work, molecular dynamics (MD) and molecular mechanics (MM) were used in conjunction with finite element simulations to study the interfacial shear strength (ISS) of a structure formed by a pristine single-walled CNT (SWCNT) inserted in a tobermorite crystal. The results show that, for a constant SWCNT length, ISS values increase when the SWCNT radius increases, while for a constant SWCNT radius, shorter lengths enhance ISS values.

## 1. Introduction

Cement and cement composites are the most used materials in the building sector due to their high compressive strength, low prices and mature production technology. However, cement also has low tensile strength, good permeability to several substances and easily cracks. To improve these weaknesses, different types of reinforcements are added to cement. Carbon nanotubes (CNTs) are one of the most promising candidates due to their excellent mechanical properties. The number of research studies on cement-CNTs composites has steadily grown in recent years [1,2,3,4,5]. CNTs have elastic moduli greater than 1 TPa and tensile strengths as high as 63 GPa [6,7]. When CNTs are homogeneously dispersed within the cement matrix, density [8], permeability [9], mechanical properties [10] and durability [11] of the composites are improved. The effect of CNTs is particularly noticeable on mechanical properties, as they can delay cracks propagation.

The CNTs—cement interface is a key factor in mechanical properties as this is typically a weak area and determines the material fracture behavior [12]. Diverse simulation methods, such as coarse-grained and all-atoms molecular dynamics [13], ref. [14] show that when CNTs are incorporated into a cement matrix, the way of fracture is changed and mechanical properties are clearly enhanced. 

Interfacial bond strength can be determined from Interfacial Shear Strength (ISS) measurements, such as fragmentation, pull-out, microdroplet, push-out and push-in tests [15,16,17,18,19,20] and most experimental studies have focused on composites made of CNTs and polymeric matrices due to the difficulties in studying CNT-cement composites. The lack of experimental information can be supplemented by the use of simulation methods such as molecular mechanics (MM) and molecular dynamics (MD). Both techniques provide atomic information about the interface, which cannot be obtained from experiments. ISS can be calculated using a pull-out model [21]. Pulling out a CNT from a matrix provide information about the interface strength. There are numerous molecular modelling studies of ISS of CNTs-reinforced polymer composites [22,23,24,25,26], while results on carbon-based reinforcements are scarce and most of them concern graphene [27,28,29,30].

The Finite Element Method (FEM) is other type of study that can be used to calculate mechanical properties of composite materials. It can be based on different formulations, such as beam models [31], representative volume element FEM analysis [3,32] or nonlinear analysis for dynamic events [33] or when crack analysis is needed [34]. 

In this work, SWCNT pull-out simulations from a tobermorite crystal were performed to calculate the ISS of several nanocomposites. MD, MM were applied to obtain interfacial energies, which were subsequently used as input in a FE calculation. Pristine SWCNTs with different radii (2.70 to 4.74 Å) and lengths varying from 9.84 Å to 19.68 Å, were inserted into a tobermorite were explained as a function of SWCNT diameter and length and related to non-bonded interactions between the SWCNT and cement. Our work aims to get more insight into the effect that SWCNT geometry plays on ISS, which is a key factor for enhancing mechanical properties of these materials. We think that the synergy achieved by using both atomistic and FE methods is a powerful way of attaining our goals.

## 2. Models and Methods

In this section, the modelling methods are described.

### 2.1. Atomistic Model Systems

**Figure 1:**
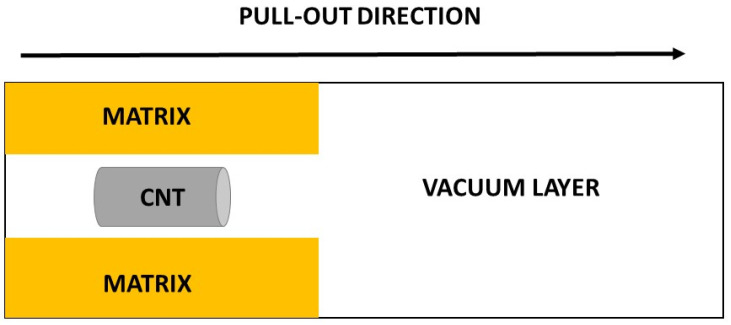
Cell used for the atomistic pull-out simulations.

The periodic simulation cell used for the pull-out atomistic calculations had dimensions of 29.54 × 67.46 × 500 Å^3^. The cell in the pull-out direction was large enough and included a vacuum layer (Figure 1).

**Figure 2:**
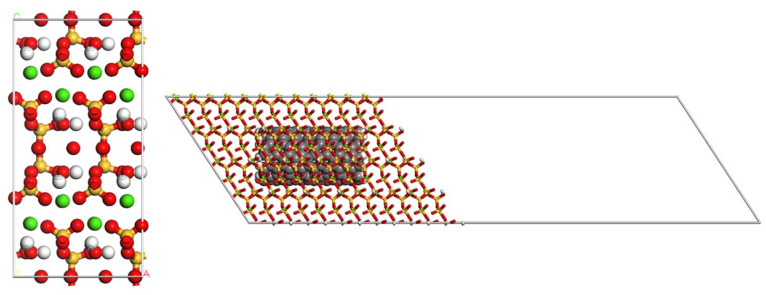
Models used in the simulation; cement: tobermorite crystal (left) and composite: tobermorite/SWCNT.

The atomic models built to represent the cement matrix and the composite material are shown in Figure 2. Despite the fact that cementitious systems are not fully crystalline [35], tobermorite 11 Å was employed to model cement by many different authors [36,37,38]. As we are mainly interested in studying interfacial interactions, crystalline tobermoritecan be a good approximation to this interface.

To build the model of the composite material, a SWCNT was inserted into the tobermorite crystal (Figure 2). Different pristine SWCNTs ((CNT (4, 4) = 2.70 Å, CNT (5, 5) = 3.39 Å, CNT (6, 6) = 4.07 Å and CNT (7, 7) = 4.74 Å) with lengths of 9.64 Å, 19.68 Å and 24.60 were used to study the influence of diameter and length on ISS. 

### 2.2. Molecular Mechanics and Molecular Dynamics

**Figure 3:**
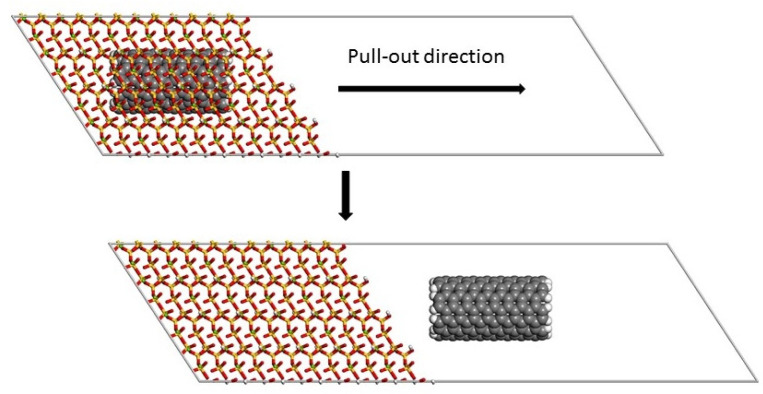
Initial and final structures before (top) and after (bottom) the SWCNT pull-out process.

The first step in creating a good model for the pull-out process was to relax the simulation lattice by means of a 100 ps simulation in the NPT ensemble at 298 K and atmospheric pressure. The temperature and pressure were controlled by a Nose-Hoover thermostat [39] and a Berendsen barostat [40] respectively. A time step of 1 fs was used during the simulation. Three independent simulations were carried out to obtain average values. After relaxing the structures, they were used to simulate the pull-out process. The size of the cell in x direction was increased and a vacuum layer was included. Before the pull-out study, these new models were subjected to a process of optimization by molecular mechanics (MM). Then, pulling-out was done by displacing the SWCNT along the x axis with increments of 10 Å. Initial and final structures before and after the SWCNT pull-out process are shown in Figure 3. After each pull-out step, the energy of the system was calculated by MM. Materials Studio 7.0 software [41] was used to perform the simulations.

The condensed-phase optimized molecular potential for atomistic simulation studies forcefield COMPASSII [42] was employed to calculate the potential energy of all systems. This forcefield has been successfully applied in numerous MD studies of systems containing CNTs and cement derived materials [28,43,44,45,46]. Coulomb interactions were calculated by the Ewald summation method while an atom-based cutoff method was applied for van der Waal interactions. The cutoff distance for both interactions was 12.5 Å.

The interfacial energy (E_interfacial_) was calculated as the energy difference between the fully embedded configuration of the SWCNT and the complete pulled-out configuration (Figure 3). This energy was then used to calculate the ISS by FEM.

### 2.3. Finite Element Method Model

A Finite Element Model based on Axisymmetric conditions has been built in MSC.Marc software by HEXAGON [47] to represent the composite material. Different FE models have been defined to cover the different combinations of radius and lengths. Same loads, boundary conditions, physical and material properties and analysis methodology are used for each geometrical combination.

#### 2.3.1. Loads & Boundary Conditions

**Figure 4:**
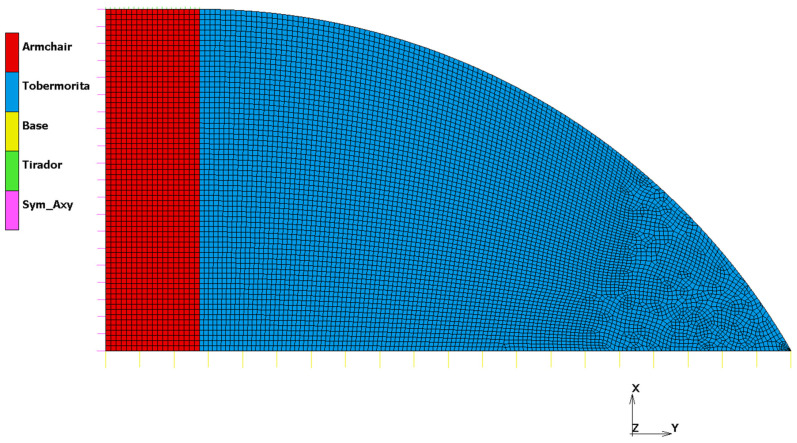
Geometry and boundary conditions for the FEM model.

Loads and Boundary Conditions were defined using contact bodies and contact interactions. Five contact bodies were defined (Figure 4): Armchair (a flexible body corresponding to Pristine armchair SWCNTs material), tobermorite (a flexible body corresponding totobermoritematrix material, Base (a rigid body defined by a curve), Tirador (a rigid body defined by a curve) and Sym_Axy (a rigid body defined by a curve). The contact interactions defined between contact bodies represented the load and boundary conditions where “Tirador–Armchair” was a glue contact interaction. This interaction defined the load as Tirador rigid body and it was controlled by its position. Tirador body was moved 1.1 × 10^−9^ mm in X-Axis, so it pulled Armchair body due to glue contact interaction. The “Sym_Axy–Armchair” contact was defined as a touching contact interaction. This interaction represents the axisymmetric boundary condition. The “Base–Armchair” pair was defined as a glue contact interaction. This interaction represented a fixed boundary condition. Finally, the “Base–tobermorite” interaction was defined as a glue contact interaction. This interaction represented a fixed boundary condition.

Furthermore, a delamination condition was defined in the interface between Armchair and tobermorite elements. This delamination condition automatically created interface cohesive elements (CZM) between both components when stresses were higher than the stablished (10^−9^ MPa). Very low values (numerically zero) were set to allow the software Marc always automatically split the mesh between Armchair and tobermorite elements, and introduce interface cohesive elements. 

#### 2.3.2. Physical Properties

Axisymmetric properties were defined for all elements of the model, for both Armchair and tobermorite components. Marc Element types 10 (Arbitrary Quadrilateral Axisymmetric Ring) and 2 (Axisymmetric Triangular Ring) were used [48].

#### 2.3.3. Material Properties

Armchair and tobermorite were defined using standard isotropic elastic material model. The Young’s Modulus (E) of the Armchair is set to 98,413 MPa and the Poisson Ratio (υ) to 0.13. The Young’s Modulus (E) of the tobermorite is set to 85,000 MPa and the Poisson Ratio (υ) to 0.3.

Interface Cohesive elements (CZM) were defined using Exponential formulation with the following properties derived from the where the values obtained with MM and MD methods in order to get a coherent traction vs opening displacement curve: Cohesive Energy (G_c_) of 9.64 × 10^−9^ mJ and Critical Opening Displacement (v_c_) of 7.81 × 10^−11^ mm.

The effective traction (t) was introduced as a function of the effective opening displacement (v) and was characterized by an initial reversible response followed by an irreversible response as soon as a critical effective opening displacement (v_c_) has been reached. The irreversible part was characterized by increasing damage ranging from 0 (onset of delamination) to 1 (full delamination). The exponential formulation has the following Equation (1) [48].

(1) $$t=G_{c}\;\frac{v}{v_{c}^{2}}e^{\frac{-v}{v_{c}}}$$

in which G_c_ is the energy release rate (cohesive energy).

#### 2.3.4. Analysis Methodology

An Implicit Non-Linear analysis using MSC.Marc is performed using a constant load step (1% of total load is applied in each load step), a convergence method based on residual forces criterion with automatic switching to displacement criterion was used, a relative convergence force tolerance of 10% was defined, large strain formulation was set and “Node-to-Segment” contact algorithm was selected.

## 3. Results and Discussion

### 3.1. Interfacial Energy

**Figure 5:**
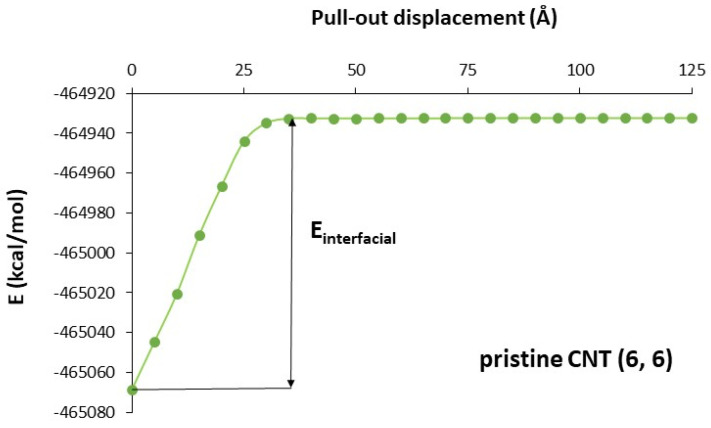
Total potential energy versus pull−out displacement.

The variation in total potential energy with pull-out displacement for the model containing a SWCNT(6, 6) is shown in Figure 5. As the SWCNT was pulled-out from the matrix, the total potential energy increased and reached a constant value beyond a certain pull-out distance. This indicated that the SWCNT and the matrix no longer interacted. The energy of the fully embedded configuration was lower than the energy of the complete pulled-out configuration. Many other authors found similar results [23,26,27,28]. The interfacial energy is the energy difference between the fully embedded configuration of the SWCNT and the complete pulled-out configuration (Figure 5).

**Figure 6:**
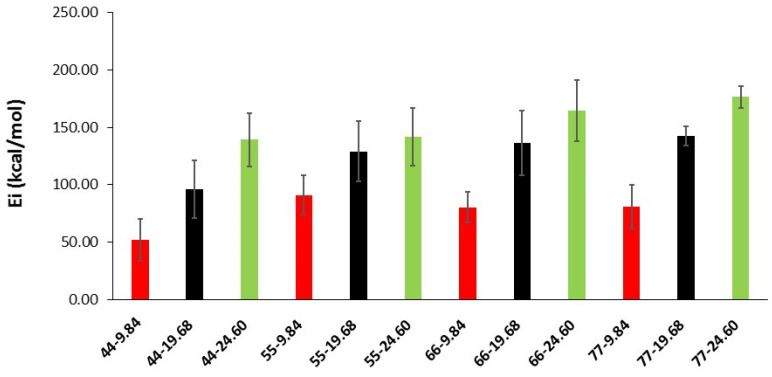
Interfacial energy as a function of SWCNT diameter and length.

The interfacial energy values are presented in Figure 6 for all systems. The standard deviation of each value is also shown in this figure. In general, interfacial energy increases when SWCNT diameter and length increase. This is probably due to the greater number of atoms in longer and bigger SWCNTs, which increases the contact points between the SWCNT and the tobermorite and, hence, the interfacial energy.

### 3.2. Interfacial Shear Strength

**Figure 7:**
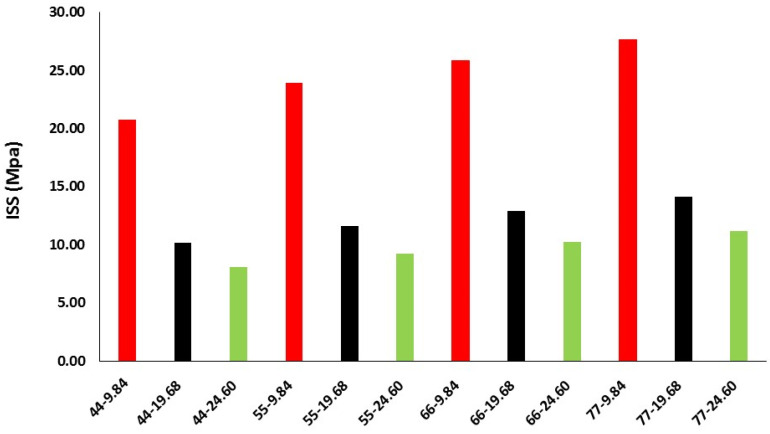
ISS as a function of SWCNT diameter and length.

For each FE model, Contact Force in X-Axis of contact body Base can be plotted with respect to position in X-Axis of contact body Tirador. The maximum value of this force can be considered as the limit value from which delamination begins to occur. This force will be used to calculate ISS dividing it by contact area between Armchair and tobermorite contact bodies (Figure 7).

ISS values are improved when SWCNT radius increases, and SWCNT length shortens. This is given by a similar extraction force (Table 1) for each diameter that leads to lower stress values when the contact area increases (length). This trend was also found by Li et al. [24] for pristine SWCNTs/polyethylene matrices. They attributed the positive correlation between SWCNT dimensions and ISS values to the increase of interfacial atoms.

It can be seen from Table 1 that, depending upon the SWCNT length and diameter, calculated ISS values ranged from 8.09 to 27.70 MPa. The effect of CNTs length on mechanical properties is controversial, as its efficiency as reinforcing agents depends on several factors such as defects on the CNT surface, concentration or dispersion in the cement matrix, although, in general, the addition of CNTs to cement leads to high improvements in mechanical properties. Some authors found that long CNTs performed better, and, to improve mechanical properties, highest amounts of short CNTs were needed [49,50].

Other authors [51] found that composites reinforced with the long CNTs showed worse properties and they attributed the efficiency of the shortest CNTs to a better filling of the nanopores within the cement matrix.

In a recent work, it was observed that the CNT length had a minimal effect on the properties of cement [52], as it is obtained in our simulations. For a given radius, the contact force can consider as constant, resulting in higher ISS for smaller areas (longer CNTs). 

Thus, to validate our approach, a comparison of our results with experimental data of single SWCNT pull-out from cement matrices would be extremely useful. To the best of our knowledge, none is currently available as the experimental characterization of these interfaces still faces numerous technical problems.

It is noticeable that, for a given radius of the SWCNT, the energy rises with the length (increase of interfacial atoms) but the ISS decreases (the contact area is higher, and the ISS is obtained by dividing the force by the area).

### 3.3. Damage Level

If a more detailed post-processing is done in the FE models, a Damage Level can be studied. Damage value indicates the amount of irreversible cohesive energy that has been lost. A value of 1.0 indicates complete loss of cohesive energy and an area where delamination will begin to occur. Damage results can be defined as follows [48]:

(2) $$D=\frac{G-G_{\mathrm{elastic}}}{G_{c}-G_{\mathrm{elastic}}};0\;\leq D\;\leq 1;v_{e}\;\geq {\;v}_{c}$$

For instance, in the case of Radius 2.71 Å and Length 9.84 Å, delamination starts when cohesive elements begin to be damaged at 24% of total load applied (Displacement × Tirador: 2.64 × 10^−10^ mm). 

**Figure 8:**
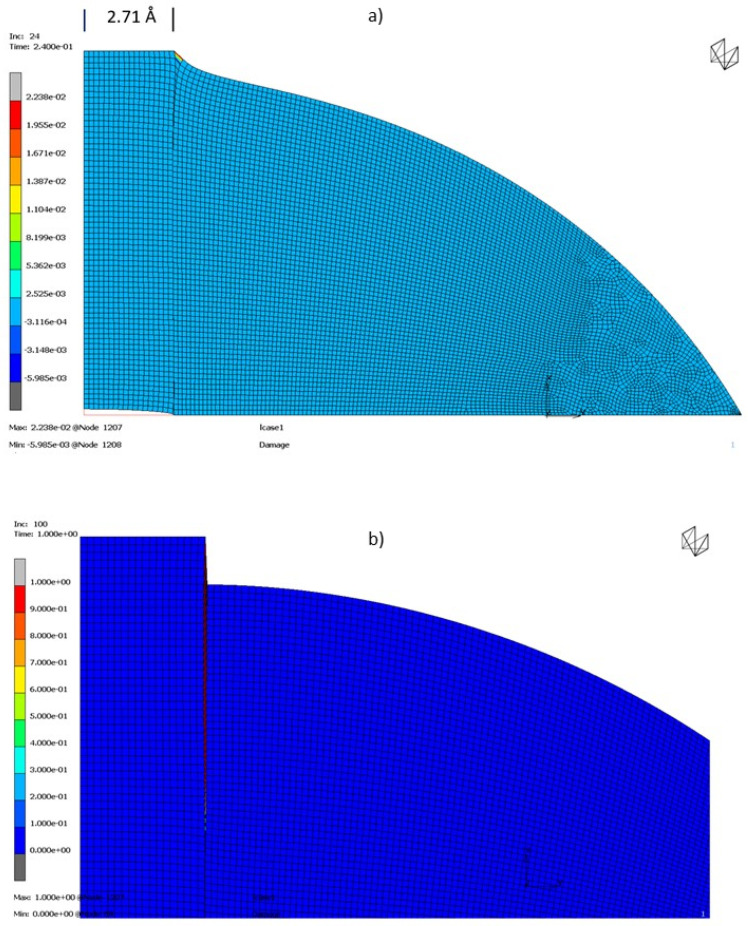
Damage level for for Radius 2.71 Å and Length 9.84 Å (**a**) Damage level when delamination starts. (**b**) Damage level when damage reaches 100%.

In Figure 8a it can be observed that crack initiates in the interface between Armchair and tobermorite when Cohesive Interface Elements begin to have irreversible behavior (2.238 % of Damage Level). It must be noticed that deformed shape is not in real scale but scaled with a factor in order to be able to see Damage Level in the Cohesive Interface Elements.

At the end of the analysis (Figure 8b)) (Displacement × Tirador: 1.1 × 10^−9^ mm), it can be observed how the delamination has grown through the interface between Armchair and tobermorite and how Damage Level of Cohesive Interface Elements have also grown until 100% of Damage Level (deformed shape is also scaled with a magnification factor).

The Damage level analysis permits to confirm that the pull-out test results are affected by the cohesion energy given by molecular models. On the other hand, the results of the test are limited by the weakest element, obtaining ISS values coherent with the cement-based materials limits [53].

## 4. Conclusions

Pristine SWCNTs with different geometrical characteristics were pulled-out from a tobermorite matrix to study the interfacial characteristics of the resulting composites by MM and MD simulations. Only non-bonded interactions between the SWCNT and the cement matrix were considered. The interfacial energy obtained from these atomistic simulations were subsequently used as input in FEM calculations to determine ISS values. ISS values increased with larger SWCNT radius, which could be attributed to a more extensive contact surface between the SWCNT and the tobermorite. However, ISS showed an opposite trend, being larger for shorter CNTs. This is due to the increased area for longer CNTs for an almost constant force for a given radius. The weakest zone of the material seems to be the tobermorite matrix. Delamination and crack growth is mainly observed at the tobermorite side of the interface. Thus, the introduction of a SWCNT of varying geometry modifies the mechanical behavior of the matrix as confirmed by the Damage Level analysis test. The pull-out results are affected by the cohesion energy, which, in turn, depends on the CNT geometry. We think the joint use of atomistic and FEM methods seems to be a good approach to describe the interfacial properties of this kind of material. It would be very interesting to be able to compare the results of this simulation with experiments, but, to our knowledge, none is currently available.

## Figures and Tables

**Table 1:** Contact force and ISS of the different systems.

Radius (Å)	Length (Å)	Maximum Contact Force (N × 10^−11^)	Contact Area (mm^2^ × 10^−12^)	ISS (MPa)
2.71	9.84	3.48	1.68	20.740
	19.68	3.40	3.35	10.158
	24.6	3.39	4.19	8.091
3.39	9.84	5.02	2.10	23.932
	19.68	4.87	4.19	11.620
	24.6	4.83	5.24	9.210
4.07	9.84	6.51	2.52	25.859
	19.68	6.49	5.03	12.896
	24.6	6.44	6.29	10.237
4.74	9.84	8.09	2.93	27.602
	19.68	8.27	5.86	14.115
	24.6	8.20	7.33	11.194

## Data Availability

All data generated and analyzed during this study are generated by commercial software and the conditions and properties are described in the article. For that reason, they are not available as supplementary information files but are available from the corresponding author on reasonable request.
